# Repeatable group differences in the collective behaviour of stickleback shoals across ecological contexts

**DOI:** 10.1098/rspb.2017.2629

**Published:** 2018-02-07

**Authors:** Jolle W. Jolles, Kate L. Laskowski, Neeltje J. Boogert, Andrea Manica

**Affiliations:** 1 Department of Zoology, University of Cambridge, Downing Street, Cambridge CB2 3DT, UK; 2 Department of Collective Behaviour, Max Planck Institute for Ornithology, Konstanz 78457, Germany; 3 Department of Biology, University of Konstanz, Konstanz 78457, Germany; 4 Department of Biology and Ecology of Fishes, Leibniz Institute of Freshwater Ecology and Inland Fisheries, Müggelseedamm 310, 12587 Berlin, Germany; 5 Centre for Ecology and Conservation, University of Exeter, Penryn TR10 9FE, UK

**Keywords:** collective behaviour, group differences, group personality, sociality, schooling, stickleback

## Abstract

Establishing how collective behaviour emerges is central to our understanding of animal societies. Previous research has highlighted how universal interaction rules shape collective behaviour, and that individual differences can drive group functioning. Groups themselves may also differ considerably in their collective behaviour, but little is known about the consistency of such group variation, especially across different ecological contexts that may alter individuals' behavioural responses. Here, we test if randomly composed groups of sticklebacks differ consistently from one another in both their structure and movement dynamics across an open environment, an environment with food, and an environment with food and shelter. Based on high-resolution tracking data of the free-swimming shoals, we found large context-associated changes in the average behaviour of the groups. But despite these changes and limited social familiarity among group members, substantial and predictable behavioural differences between the groups persisted both within and across the different contexts (group-level repeatability): some groups moved consistently faster, more cohesively, showed stronger alignment and/or clearer leadership than other groups. These results suggest that among-group heterogeneity could be a widespread feature in animal societies. Future work that considers group-level variation in collective behaviour may help understand the selective pressures that shape how animal collectives form and function.

## 1. Introduction

A fundamental goal in biology is to understand how animal collectives and societies form and function [1]. Considerable computational [1–3] and experimental work [4–6] has shown that the seemingly complex collective behaviour of animal groups can often be explained by relatively simple interaction rules [3,5,6]. By predominantly focusing on average group-level patterns and assuming animal groups to be homogeneous (but see, e.g. [2,7]), this research has proved invaluable in revealing the general principles of collective behaviour [1]. However, recent studies have started to increasingly consider the role of heterogeneity within groups, showing that even small individual differences can have large consequences for collective behaviour [2,8–10]. Besides individual differences in state (e.g. body size or energy levels), consistent behavioural variation among animals (‘animal personalities’, [11,12]) has been shown to play a fundamental role in many collective processes, such as leadership [10,13–16], social network dynamics [17,18], group exploration [19] and group functioning [10,20–22].

Despite increased attention for *within*-group heterogeneity, little is still known about *among*-group heterogeneity and whether groups themselves may differ consistently from each other, a phenomenon sometimes referred to as ‘collective personalities’ [23–25]. The few studies on this topic have mostly considered species living in highly structured and stable groups of related individuals, such as colonies of social insects and spiders [24]. For example, Wray *et al.* [23] and Pruitt *et al.* [26] demonstrate that consistent differences exist in the collective defensiveness and foraging behaviour of colonies of honeybees and social spiders. Social arthropods are unique among grouping animals by showing high relatedness, often coupled with high behavioural specialization and extreme fidelity to their group [24,27]. However, many social animals experience far more fluid social structures where group membership changes frequently over time and individuals are less related to each other. If group-level behavioural patterns can emerge from the characteristics of individuals within groups, as predicted by theory [2,28,29] and shown mechanistically by recent experimental and observational work [7,10], consistent behavioural differences between groups should emerge even without high relatedness or extensive social experience among group members, as in the case of animal fission–fusion societies.

The behaviour and structure of animal groups may fluctuate considerably even when the individuals in that group use identical interaction rules [30]. In particular, collective behaviour may change considerably depending on the context. For example, groups of animals may become more cohesive with increasing predation risk but scatter when foraging [31,32]. Many animal species live in spatially complex environments where the appearance and persistence of food, shelter and predators can be patchy [33,34]. This raises the question of whether consistent behavioural differences between groups would persist across different ecological contexts that may potentially result in considerable changes in average group behaviour. Such cross-context behavioural consistency between groups may have large effects on their functioning and performance, such as the ability to locate food or avoid predators, and thereby affect the fitness of individuals within those groups [8,10]. Most studies on collective behaviour have focused on a single biological context [30], and experimental tests of how the environmental context may result in group-level changes in behaviour have been limited (e.g. [35,36]). Whether animal groups exhibit repeatable behavioural differences across ecological contexts thus remains an open question.

Here, we studied the fine-scale movements and structure of freely moving groups of wild-caught sticklebacks (*Gasterosteus aculeatus*) to investigate the possibility that consistent and predictable group differences emerge and are maintained across different contexts. We exposed shoals to three ecologically relevant environments: a large, open tank without food or cover (open context), an open tank with patches of food (foraging context) and an open tank containing food patches and plant cover (cover context). To limit social experience among group members, which could potentially drive consistent differences among groups (e.g. [36]), we assigned individuals to groups randomly and allowed fish to interact with their group mates only during the experimental trials. We filmed the groups from above and used custom-tracking software to acquire high-resolution movement data for each fish which we used to compute key characteristics of animal groups that may affect group functioning in terms of anti-predator, foraging and locomotor benefits: group speed, cohesion, alignment and leadership structure [1,33,34]. We then adapted the mixed-modelling approach from the personality literature [37,38] to test whether and how (i) these group behaviours changed, on average, across the different ecological contexts and (ii) behavioural differences among groups were maintained within and across these contexts.

## 2. Material and methods

### (a) Subjects

We collected three-spined sticklebacks (*G. aculeatus*) with dip nets from a stream near Cambridge, England, and transferred them immediately to our fish facilities at the University of Cambridge (temperature: 14 ± 1°C; light/dark: 12 /12 h). Fish were housed socially in large glass tanks (120 cm length × 60 cm width × 60 cm height) that contained artificial plants and shelters. We fed fish defrosted bloodworms (*Chironomid* larvae) *ad libitum* once daily. After six months, we haphazardly selected 125 individuals (controlling for body size: 40.6 ± 0.6 mm) from the social housing tanks. As it is impossible to accurately sex sticklebacks non-invasively, our test subjects were assumed to be of mixed sex. As we kept sticklebacks' controlled light and temperature conditions corresponding to the non-breeding season, during which both sexes are non-territorial and actively shoal together, sex differences in behaviour are assumed unlikely to play a prominent role.

### (b) Experimental procedure

At the start of the experimental period, fish were moved to experimental housing tanks (80 cm × 20 cm × 18.5 cm) and allocated to individual compartments to minimize potential social modulation and acclimatization effects [39]. Each experimental housing tank consisted of eight individual holding compartments (9.5 × 20 × 18.5 cm) that each contained a gravel substrate and an artificial plant. To reduce the potential stress of social isolation, compartments were separated by perforated transparent partitions that allowed the transfer of visual and chemical cues between fish. We haphazardly allocated fish to 25 groups of five while ensuring that individuals assigned to the same group were never housed in the same experimental holding tank. Before the group assays, fish were tested individually in classic boldness and sociability assays of which the data are explored in detail in another paper [10]. To enable individual identification of the fish, 2 days before the start of the collective behaviour experiments, we tagged all fish on their middle dorsal spine with a coloured disc-shaped tag (5 mm diameter) made from electrical tape. These tags have no major influence on the activity and shoaling behaviour of three-spined stickleback [40].

### (c) Group assays

**Figure 1.**
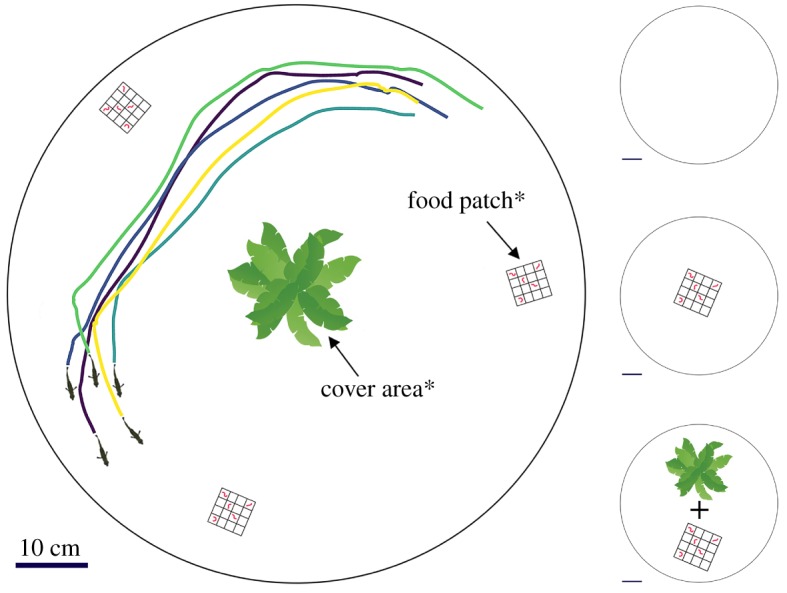
Schematic of the tank in which the groups of fish were tested across three different contexts: (i) the open context, an environment without food or plant cover, (ii) the foraging context, an environment with three patches of food* and (iii) the cover context, an environment with food patches* as well as plant cover*. Tracking segments are shown for one randomly selected group.

We repeatedly exposed each of the 25 groups to three ecologically relevant contexts: an open-field environment (open context) where the tank contained no food or shelter [30], a foraging environment where the tank contained several patches of food (foraging context) and an environment that included food patches as well as a refuge in the form of artificial plants (cover context). We used two white, circular Perspex tanks (80 cm diameter, 20 cm height; 7 cm water depth; figure 1) positioned inside a large white light tent (200 × 100 × 160 cm) to reduce potential external disturbances while facilitating consistent diffuse lighting by halogen lamps outside the tent. At the start of a trial, fish were transferred from their individual compartment to black plastic cups and allowed to acclimatize for 30 s. Subsequently, all five fish in a group were simultaneously placed in a transparent Perspex cylinder (10 cm diameter) in the centre of the tank. After 30 s, the cylinder was raised remotely and the trial was started and at the end of the trial, fish were put back in their individual holding compartments. Fish thus only experienced their group mates during the experimental trials. All groups were tested in water that was used previously for other groups. Each trial we made sure to remove any excrement and uneaten food items from the test tank and thoroughly mixed the tank's water to diffuse any remaining chemical cues.

We first exposed groups to the open context, where the tank contained no food or refuge. This is the classic test environment for studying free-schooling dynamics [30]. Groups were tested in this context for 30 min once as we were interested in group exploration of a novel environment. After the trial in the open context, and thus having had time to become familiar with the testing environment, we exposed the groups over the next 2 days to the foraging context. We added three food patches, a white opaque plastic lattice of 5 × 5 × 1 cm containing 16 squares, to the circular testing environment in roughly equilateral triangular formation. Each food patch contained five bloodworms and was noticeable by the fish from a distance of approximately 10–15 cm. Each group received four 5-min trials in this context: one trial in the morning and one in the afternoon on 2 consecutive days. To have data of comparable length to the open context trials, we only looked at the first 5 min of the data in that context. Finally, we exposed the groups to the cover context. Here, in addition to the three food patches, we also provided artificial plants (15 cm diameter) in the centre of the test tank. Each group received two 10-min trials in the morning on 2 consecutive days in this cover context. Groups received multiple trials in the two foraging contexts to acquire sufficient foraging data (explored in [10]) and trials in the cover context were run for longer as the availability of cover resulted in fish spending considerably less time exploring the tank. We tested the groups in a randomized order within each context to reduce the potential of time-of-day effects. We used a fixed testing order of contexts such that all groups experienced the open context first, followed by the foraging context, and finally the cover context. The rationale of this testing order was to first test the fish in a novel open environment (open context) and to only add new features after the fish were familiar with the environment, and sequentially (i.e. first food, then cover) to avoid fish searching for them following their removal. At the end of each day, fish were fed three bloodworms in their individual home compartments.

### (d) Data collection

We recorded all trials with Raspberry Pi 2 Model B computers (RS Components Ltd) and associated cameras (Camera Module V1, RS Components Ltd) positioned above the test tanks. From the videos, we automatically identified fish based on their differently coloured tags and acquired highly detailed individual-based movement data using custom-written tracking software (AnimTrack by J.W.J.). Positional coordinates were converted from pixels to millimetres and subsequently smoothed using a Savitzky and Sgolay smoothing filter with a window of 15 frames. After tracking, all trajectory data were checked visually and, in case of missing data or tracking errors, manually corrected. Using the tracking data from the individuals in each group, we determined the group centroid and calculated several key characteristics of individual behaviour within groups: individual swim speed, individual distance to the group centre (cohesion) and proportion of time individuals spent in front of the group centroid (leadership). We focused on these individual measures as how fast individuals swim as a group, how closely they stick together and their positions within the group should strongly influence the overall behaviour and function of the group, including their ability to find novel foraging areas, the ability to detect and escape from predators, as well as the transfer of social information [10,33,34,41]. We additionally computed two characteristics of the emergent group behaviour as a whole: polarization, which is a measure of the alignment of the fish in the group relative to each other that ranges from 0 (complete non-alignment) to 1 (complete alignment) [3] and variance in the proportion of time fish spent in front of the group centre (leadership structure). Details of the computation of these behavioural measures can be found in the electronic supplementary material.

### (e) Data analysis

Our main goal was to investigate cross-context consistency in group behaviours as we expect such group-level consistency to have broadest ecological and evolutionary ramifications. However, before addressing this, we first ascertained that the groups exhibited consistent behavioural differences within each of the contexts in which they were repeatedly tested (foraging and cover contexts). Therefore, we ran separate linear mixed models for each of the individually measured behaviours (individuals' median speed, mean distance to the group centre and mean proportion of time in front of the group centroid), and in each context (foraging and cover) with trial added as a fixed factor, and Group ID and Individual ID added as random factors. In the foraging context, we only focused on behaviour that occurred before the food was depleted. As the open context only contained one trial, we could not investigate within-context repeatability for this context.

Next, we investigated across-context repeatability in group behaviour. We focused our analyses on the first trial in each context as we were particularly interested in the groups’ behaviour upon initial exposure to the environment, thereby also avoiding potential acclimatization and satiation effects. We included context as a fixed factor, where we divided the foraging context into the time before and after food depletion, giving a four-level factor for test context. We ran separate models with individuals' median speed, mean distance to the group centre and mean proportion of time in front of the group centroid fitted as response variables, and Group ID and Individual ID added as random factors. In addition, we ran models with median group polarization and group variance in the proportion of time fish spent in front of the group centre (i.e. variance in leadership) as group-level response variables, context as a fixed factor and group ID as a random effect. Running the models including all trials in all contexts, rather than the first trial only, did result in qualitatively similar results (electronic supplementary material, table S1).

We used general linear mixed models with Gaussian error distributions and Markov chain Monte Carlo (MCMC) estimation for all analyses using the MCMCglmm package in R [42], which returns 95% credibility intervals (CIs) of the parameter estimates for fixed effects and variance estimates for random effects. If the 95% CIs of two parameter estimates did not overlap, we interpreted this as evidence that the estimates were significantly different from each other; if the 95% CI of a random effect did not reach zero, this was interpreted as evidence for significant repeatability. From the models we calculated within- and across-context group and individual repeatability (‘consistency repeatability’ *R*_C_, [38]) based on the proportion of variance explained by respectively group and individual identity relative to the total variance. Throughout, we used non-informative proper priors. Preliminary analyses indicated that our results were not sensitive to changes in prior settings (results not shown). We compared the posterior distributions and auto-correlation plots of five independent chains with 500 000 iterations with a burn-in period of 5000 and a thinning interval of 100 to ensure convergence and adequate chain mixing (see [42] for details). For analyses of the cover context trials, we conducted analyses both on data from the entire trial and on only the time during which all five fish of the group were out of cover. There was no qualitative difference in the patterns of across-group repeatability with the subsetted dataset (electronic supplementary material, table S2).

## 3. Results

### (a) Context effects on collective behaviour

**Figure 2.**
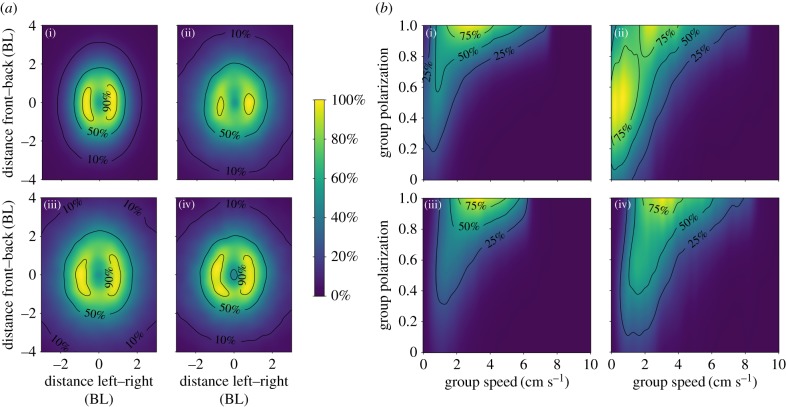
Heat maps depicting (*a*) the probability of finding neighbouring fish at a given position relative to the position of the focal fish placed at the origin pointing up, with distances expressed in units body length (BL), and (*b*) the relationship between group speed (mean individual speed in the group) and polarization (alignment among all group members). Panels represent the open context (i), the foraging context until all food was depleted (ii), the foraging context after all food was depleted (iii) and the cover context (iv). Plots were calculated based on a frame-by-frame basis with time steps of 1/24th s and are presented in percentages relative to the densest bin for that context. These figures show that (*a*) neighbouring fish could primarily be found within two body lengths side-by-side and four body lengths front-to-back, with groups being most cohesive in the open context, and (*b*) that there was a strong positive relationship between the speed and polarization of the groups that was not itself strongly affected by the context.

**Figure 3.**
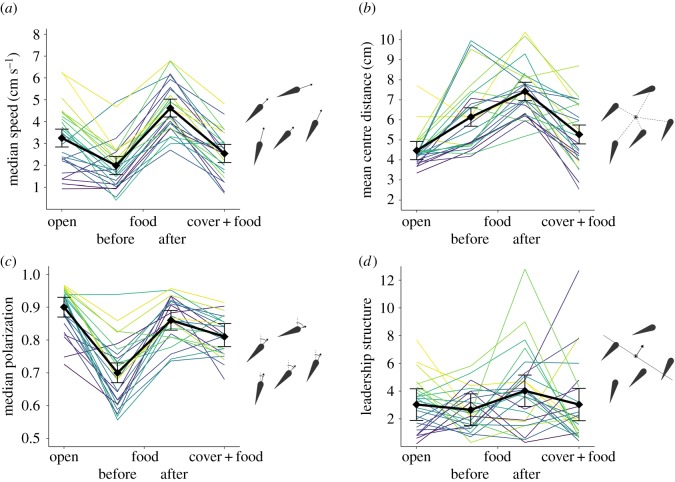
Line plots of group behaviour across the three contexts, with the foraging context split into time before and after food was depleted: (*a*) average individual median speed in the group, (*b*) mean group centre distance, (*c*) median polarization and (*d*) leadership structure, the variance in the proportion of time group members were in front of the group centre. Lines indicate groups, with colours indicating group rank in terms of the group's behaviour in the open context. Black diamonds indicate average group behaviour per context with error bars indicating 95% creditability intervals in terms of certainty of the mean extracted from the MCMCglmms. Non-overlapping confidence intervals indicate a significant difference.

On average, the groups changed their behaviour considerably across the different contexts (see figures 2 and 3). In the open context, the shoals swam at a moderate speed (median: 3.25 cm s^−1^, 95% confidence interval: [2.84–3.66]), were highly cohesive (mean distance to centre: 4.47 [4.01–4.93] cm), were strongly aligned (median polarization: 0.90 [0.87–0.93]) and showed a clear leadership structure (variance in proportion of time fish spent in front of the group: 3.0 [1.9–4.2] × 10^−2^), i.e. some groups had clearer differences in leader–follower positioning among its members than other groups.

Compared with the open context, in the foraging context before the food was depleted (figure 3), groups moved at a significantly lower speed (median: 1.99 [1.58–2.40] cm s^−1^), were less cohesive (mean distance to centre: 6.14 [5.67–6.60] mm) and showed lower alignment (median polarization: 0.70 [0.67–0.73]). After the food was depleted, groups increased their speed considerably (median: 4.62 [4.21–5.03] cm s^−1^) and further decreased their cohesion (mean: 7.41 cm [6.94–7.88]). Furthermore, the alignment of the group increased considerably (median polarization: 0.86 [0.83–0.89]) and became as high as in the open context. Leadership structure (variance in the proportion of time spent in front of the group) was not significantly affected by the presence (2.6 [1.5–3.8] × 10^−2^) or subsequent depletion of food (4.0 [2.9–5.2] × 10^−2^).

Finally, the availability of shelter in the environment resulted in fish forming smaller shoals and groups to often split. On average, 2.58 fish (range: 1.04–4.37) were out of cover together at any one time, while all fish were out of cover simultaneously only 30.0% of the time (range: 0–73.2%). The speed (median: 2.54 [2.13–2.96] cm s^−1^), cohesion (mean distance to centre: 5.27 [4.79–5.74] cm) and alignment (median polarization: 0.81 [0.78–0.85]) were at an intermediate level between the open and foraging contexts. When all fish were out of cover simultaneously, they moved at a significantly higher speed (median: 3.41 [3.14–3.98] cm s^−1^) and were less cohesive (mean individual centre distance: 66.4 [61.6–71.2] mm) compared with when only a few fish were out. Leadership structure was similar compared with the open context (3.0 [1.9–4.2] × 10^−2^).

### (b) Within-context group repeatability in collective behaviour

The shoals of randomly grouped, unfamiliar sticklebacks exhibited significant repeatable differences in behaviour within the foraging and cover contexts, with group behaviour being significantly repeatable in terms of swimming speed (foraging context: *R*_C_ = 0.55; cover context: *R*_C_ = 0.45), cohesion (*R*_C_ = 0.42; *R*_C_ = 0.47), alignment (*R*_C_ = 0.32; *R*_C_ = 0.24) and leadership structure (*R*_C_ = 0.42; *R*_C_ = 0.40) (see the electronic supplementary material, tables S3 and S4 for details). No within-context repeatability could be measured for the open context as fish were only tested only for their response to the novel (open) environment.

### (c) Across-context group repeatability in collective behaviour

Despite the large context-associated changes in the average behaviour of the groups, behavioural differences among groups were maintained across the three different contexts (table 1 and figure 3): group behaviour was significantly repeatable in terms of swimming speed (*R*_C_ = 0.49), cohesion (*R*_C_ = 0.21), alignment (*R*_C_ = 0.29) and leadership structure (*R*_C_ = 0.29). Also within the groups, there were repeatable differences between the individual fish in terms of their average distance from the group centre (*R*_C_ = 0.19) and the proportion of time they spent in the front of the group (*R*_C_ = 0.24). However, fish strongly conformed in their speed within a group. As a result, there was very low variance in speed among them relative to the large differences in speed between groups and, consequently, in a lack of individual repeatability in speed.

**Table 1.** Variance and repeatability in group (gr) and individual (id) behaviour across the contexts. Data show values with 95% creditability intervals (CI) from the MCMC glmm models. If the 95% CIs of the random effect did not overlap with zero, this was interpreted as evidence for significant repeatability.

	Var group level	Var individual level	Var residual	*R*_C_ group	*R*_C_ individual
gr speed	0.85 [0.49–1.64]	0.00 [0.00–0.00]	0.87 [0.77–1.00]	0.49 [0.39–0.62]	0.00 [0.00–0.00]
gr centre distance	0.75 [0.33–1.65]	0.67 [0.36–1.10]	2.12 [1.84–2.47]	0.21 [0.13–0.32]	0.19 [0.14–0.21]
gr polarization	1.70 [0.62–4.10] × 10^−3^	—	4.18 [3.05–5.90] × 10^−3^	0.29 [0.17–0.41]	—
gr leadership structure	2.32 [1.20–4.92] × 10^−4^	—	5.78 [4.31–7.99] × 10^−4^	0.29 [0.22–0.38]	—
id leadership	—	0.62 [0.36–0.96] × 10^−2^	2.00 [1.73–2.32] × 10^−2^	—	0.24 [0.17–0.29]

## 4. Discussion

Using detailed individual-based tracking of free-swimming stickleback shoals, we found that groups changed their behaviour considerably depending on the environment. In an open context without food or cover, groups tended to move at a moderate speed, had high alignment and were relatively cohesive. The addition of food led groups to move more slowly while becoming less cohesive and less aligned. Finally, an environment with both food and plant cover resulted in often only subsets of fish to emerge that were generally more cohesive and well aligned. However, adapting the statistical framework used in the animal personality literature [37,38] to investigate consistent behavioural differences at the level of the group, we demonstrate that groups had consistent behavioural differences that persisted not only within but also across different contexts: some groups were consistently faster, more cohesive, more aligned and/or exhibited clearer leadership structure than other groups. These differences arose despite individuals being randomly allocated to groups and only experienced each other during the experimental trials.

Our results show that consistent group-level differences can exist even in species that do not exhibit the high relatedness and strong social structures of social insects [23–26]. Here, we tested randomly composed groups of which the group members had no social contact in the weeks preceding the experiments and could only interact during the test trials. Furthermore, all fish were of similar size, received the same amount of food, came from the same wild population and were pseudo-randomly (controlling for size) selected from a sample of approximately 500 fish housed in large social housing tanks. It is therefore unlikely that the large and consistent group differences we observed are the result of either high social familiarity or relatedness between the individuals within the groups. Although the composition of the groups remained the same throughout the experiment, group members only had very limited time to interact with each other. Repeated social interactions are therefore also unlikely to be an important explanatory factor. Rather, our results suggest that social feedback arising from the interactions among individuals can be very rapid (see also [14,43]) and important in driving collective behavioural patterns and differences among groups. Indeed, in a complementary paper on the same dataset in which we investigated the behavioural tendencies of the fish, we found that heterogeneity in terms of those differences among the groups explained their emergent collective behaviour within each of the contexts [10]. These results together highlight that group differences in collective behaviour can emerge rapidly from the characteristics of individuals within groups and that such group-level differences remain consistent even across different contexts that elicit considerable changes in average group behaviour.

Little is still known about the formation and stability of animal groups in wild populations of many social species, especially non-terrestrial mammals. Wild stickleback shoals are relatively fluid and individuals interact with lots of different partners, but nevertheless tend to associate repeatedly with certain conspecifics [44]. Furthermore, behavioural differences have been observed among wild stickleback groups [45]. In the case that groups behave differently from one another in a consistent manner, as we show in this paper, this may have large effects on their relative performance, such as their ability to locate food or avoid predators, and thereby affect the fitness of individuals within those groups [8,10]. In turn, such group-dependent performance may, along with passive assortment effects [3], influence how individuals associate with one another in populations and determine the formation of animal groups [8,46]. Hence, integrating heterogeneity within and among groups in both field studies and laboratory experiments on the composition and stability of animal groups may help to understand the selective pressures that have shaped social behaviours. Such an understanding may potentially also provide insights into the collective functioning and performance of human social groups, a topic of considerable interest in the human literature [47,48].

Beyond consistent behavioural differences between the groups, we found that, on average, all groups changed their behaviour considerably depending on the ecological context. Most work on collective behaviour has been conducted on animals in a single biological context (but see, e.g. [34,49]), in particular in a large open, homogeneous environment [30]. Here, we show that in the presence of food, groups slowed down and became less cohesive but that after food was depleted groups increased their speed even beyond that observed in the non-foraging context and became more spread out. This is largely consistent with previous research, which shows that in the presence of food, fish move faster and form looser schools [31,32,50,51], with fish slowing down when they are actually feeding. In a foraging context, fish may swim in looser shoals as competition may be higher and because fish may forage less efficiently when in tight shoals [50]. Although changes in group cohesion are closely linked with, and can emerge from, differences in speed [10], our current results show that the cohesion between individuals is also affected directly by the availability of food. Enhanced familiarity with the environment due to repeated testing may have resulted in some further increases in speed when the food was depleted. When plant cover was available, individuals spent considerable time hiding under cover and often only a subset of individuals emerged (on average half the group). This is likely linked to the small group size used, as hiding under cover is a more effective means of predator avoidance than grouping when there are few conspecifics [52]. When fish were out of cover, thus often with only few individuals, they grouped more closely together and showed more synchronized movements than in the foraging context without cover. This finding could potentially be explained by individuals in small groups perceiving a relatively higher risk of predation, experiencing lower competition and having higher foraging efficiency than when in larger groups, which thereby increases the relative benefits of group cohesion and alignment. Indeed, Ioannou *et al.* [53] showed that highly coordinated groups of simulated prey were less at risk than their counterparts in unpolarized groups. Furthermore, Schaerf *et al.* [32] found that groups of fish slowed down and contracted when they experienced predator cues. These results together highlight how animals trade-off the opposing forces of grouping and how that depends on the context, with on the one hand protection from predation and facilitation in finding food and on the other hand increased competition and decreased foraging efficiency [33,50].

The last two decades have seen a shift from understanding general patterns in animal behaviour to behavioural differences between individuals, resulting in a well-established literature that shows consistent behavioural differences are ubiquitous [11], have major ecological and evolutionary implications [54,55] and, increasingly, play a fundamental role in the functioning of animal groups [10,13–22,56]. Here, we show that stable differences also exist between groups even without long-term associations among group members and that these group differences can be maintained across different ecological contexts that considerably change average group behaviour. Ultimately, such consistent differences between groups could potentially affect the survival and reproductive success of the individuals within them and likely influence how individuals associate with one another. Hence, an increased consideration of the causes and consequences of heterogeneity not only within but also between groups may further our understanding of the selective pressures that have shaped social behaviours. Ultimately, this may help build a more complete picture of how animal collectives form and function.

## Supplementary Material

Supplementary methods/Supplementary tables

## Data accessibility

Data are deposited in the Cambridge data repository (doi:10.17863/CAM.12136) [57].
