# Supplementary material for: Repeatable group differences in the collective behaviour of stickleback shoals across ecological contexts
Source: Proc Biol Sci. 2018 Feb 7;285(1872):20172629. doi: 10.1098/rspb.2017.2629 (PMC5829202; doi:10.1098/rspb.2017.2629)
Supplement: Supplementary methods/Supplementary tables [file rspb20172629supp1.pdf]

ELECTRONIC SUPPLEMENTARY MATERIAL

For:

Repeatable group differences in the collective behaviour of stickleback shoals across ecological contexts

Jolle W. Jolles, Kate L. Laskowski, Neeltje J. Boogert, and Andrea Manica

SUPPLEMENTARY METHODS

Quantification of behavioural data

Frame-by-frame computations

From the discrete tracking data, we determined each fish's velocity, speed, direction, and relative position to the group centroid over time using the following series of calculations. With the vector $\mathbf{r}_i(t) = (x_i(t), y_i(t))$ denoting the position of fish i at time t, we approximated its velocity $\mathbf{v}_i(t) = (u_i(t), w_i(t))$ using the forward finite difference

$$\mathbf{v}_i(t) = \frac{\mathbf{r}_i(t + \Delta t) - \mathbf{r}_i(t)}{\Delta t}, \tag{1}$$

where Δt = 1/24 s is the time interval between subsequent position measurements. The speed $\mathrm{v}_i(t)$ is then given by the norm of the velocity vector, such that

$$\mathrm{v}_i(t) = |\mathbf{v}_i(t)| = \sqrt{u_i{}^2(t) + w_i{}^2(t)}. \tag{2}$$

Next, we quantified the direction of motion using the angle $\psi_i(t)$ between the velocity vector and the positive y-axis, which is given by

$$\psi_i(t) \; = \; atan2(w_i(t), u_i(t)). \tag{3}$$

Subsequently, we identified the mean coordinates of all fish in a group (n) as $\mathbf{r}_c(t) = (x_c(t), y_c(t))$, that is, the group centroid ('centre'), and, in the same way as for the individual fish (equation 3), calculated the group centre's direction of motion $\psi_c(t)$. Subsequently, we calculated the distance of each fish to the group centre ('center distance')

$$CD_i(t) = \sqrt{(x_i(t) - x_c(t))^2 + (y_i(t) - y_c(t))^2}. \tag{4}$$

To calculate relative positions of the fish in a group, we shifted the coordinates of each fish so that the origin of the coordinate system was at the group centroid. We then determined the angle between the positive y axis through the group centroid and an individual's position as

$$\delta_i(t) = \; atan2\big(x_i(t) - x_c(t), \; y_i(t) - y_c(t)\big), \tag{5}$$

which we used to calculate an individual's relative direction to the direction of the group centre

(6)

$$\sigma_i(t) = \delta_i(t) - \psi_c(t),$$

which we then adapted to fit in the cartesian coordinate system pointing north

$$\{\sigma_i(t) < -\pi\}: \sigma_i(t) = 2\pi - |\sigma_i(t)| \text{ or } \{\sigma_i(t) > \pi\}: \sigma_i(t) = -\big(2\pi - \sigma_i(t)\big). \qquad (7)$$

Based on the relative direction and distance to the group centre, we calculated the relative position for each fish in a group to the group cenroid

$$(x_i', y_i') = CD_i(t)\big(\sin\big(\sigma_i(t)\big), \cos\big(\sigma_i(t)\big)\big). \qquad (8)$$

These transformed coordinates enabled us to determine spatial leadership positioning of the fish, with a positive y' indicating a fish was in front and a negative y' indicating a fish was behind the group centroid. Finally, for each frame we calculated the polarisation of the group

$$\rho(t) = \frac{1}{n}\sqrt{\Big(\sum_{i=1}^{n} sin(\psi_i(t))\Big)^2 + \Big(\sum_{i=1}^{n} cos(\psi_i(t))\Big)^2}, \qquad (9)$$

which is a measure of the alignment of the fish in the group relative to each other and ranges from 0 (complete non-alignment) to 1 (complete alignment).

Behavioural scores

For each trial, we calculated 1) fish's median speed (cm/sec), 2) fish's mean distance to the group centre (mm), 3) fish's proportion of time spent in front of the group centre ('leadership'), 4) variance in group member's proportion of time spent in front of the group centre ('leadership structure'), and 5) the median polarisation of the group ('alignment'). We calculated variance in leadership as we were interested in how clear the individuals in a group differed in their tendency to be in front or back of the group. If all individuals were randomly positioned, their average front-back position would be close to zero and hence result in a very low group-level variance. In addition, for certain plots (Figure 3 in the main text) we calculated the mean of the group members' median speed over time and the mean of the group members' distance to the group centroid. Mean values were calculated for approximately normal (transformed) distributions and median values when data was skewed.

ELECTRONIC SUPPLEMENTARY MATERIAL

For:

Repeatable group differences in the collective behaviour of stickleback shoals across ecological contexts

Jolle W. Jolles, Kate L. Laskowski, Neeltje J. Boogert, and Andrea Manica

SUPPLEMENTARY TABLES

Table S1. Variance and repeatability in group (gr) and individual (id) behaviour across all contexts including all trials.

	Var group level	Var individual level	Var residual	R_C group	R_C individual
gr speed	0.67 [0.38 – 1.28]	0.08 [0.03 – 0.14]	1.03 [0.96 – 1.12]	0.37 [0.28 – 0.50]	0.04 [0.03 – 0.05]
gr centre distance	0.86 [0.42 – 1.81]	0.78 [0.54 – 1.34]	2.72 [2.51 – 2.95]	0.20 [0.12 – 0.31]	0.18 [0.16 – 0.19]
gr polarisation	1.11 [0.51 – 2.47] × 10^{-3}	-	5.00 [4.21 – 6.00] × 10^{-3}	0.18 [0.11 – 0.29]	-
gr leadership structure	1.60 [0.90 – 3.15] × 10^{-4}	-	6.01 [5.06 – 7.21] × 10^{-4}	0.21 [0.15 – 0.30]	-
id leadership	-	0.82 [0.60 – 1.11] × 10^{-2}	1.97 [1.82 – 2.13] × 10^{-2}	-	0.29 [0.25 – 0.34]

Data show values with 95% creditability intervals (CI) from the MCMCglmm models. If the 95% CIs of the random effect did not overlap with zero, this was interpreted as evidence for significant repeatability.

Table S2. Variance and repeatability in group (gr) and individual (id) behaviour across all contexts based on only the first trial in each context and with data from the cover context subsetted to only the time when all fish were out of cover simultaneously.

	Var group level	Var individual level	Var residual	R_C group	R_C individual
gr speed	0.86 [0.49 – 1.67]	0.00 [0.00 – 0.00]	0.88 [0.77 – 1.01]	0.49 [0.39 – 0.62]	0.00 [0.00 – 0.00]
gr centre distance	0.67 [0.26 – 1.51]	0.84 [0.50 – 1.34]	2.06 [1.78 – 2.41]	0.19 [0.10 – 0.29]	0.24 [0.20 – 0.25]
gr polarisation	1.57 [0.52 – 3.95] × 10^{-3}	-	4.57 [3.31 – 6.52] × 10^{-3}	0.26 [0.13 – 0.38]	-
gr leadership structure	2.32 [1.21 – 4.94] × 10^{-4}	-	5.78 [4.32 – 7.97] × 10^{-4}	0.29 [0.22 – 0.38]	-
id leadership	-	0.62 [0.37 – 0.97] × 10^{-2}	1.90 [1.63 – 2.21] × 10^{-2}	-	0.25 [0.18 – 0.30]

Data show values with 95% creditability intervals (CI) from the MCMCglmm models. If the 95% CIs of the random effect did not overlap with zero, this was interpreted as evidence for significant repeatability.

Table S3. Variance and repeatability in group (gr) and individual (id) behaviour across all four trials of the foraging context before food was depleted.

	Var group level	Var individual level	Var residual	R_C group	R_C individual
gr speed	0.94 [0.55 – 1.81]	0.00 [0.00 – 0.00]	0.76 [0.67 – 0.87]	0.55 [0.45 – 0.66]	0.00 [0.00 - 0.00]
gr centre distance	1.94 [1.04 – 3.92]	0.79 [0.49 – 1.23]	1.85 [1.61 – 2.15]	0.42 [0.33 – 0.54]	0.17 [0.15 - 0.17]
gr polarisation	3.03 [1.09 – 7.05] × 10^{-3}	-	6.47 [4.71 – 9.25] × 10^{-3}	0.32 [0.19 – 0.43]	-
gr leadership structure	2.00 [1.09 – 3.99] × 10^{-4}	-	2.80 [2.07 – 3.90] × 10^{-4}	0.42 [0.34 – 0.51]	-
id leadership	-	0.87 [0.61 – 1.24] × 10^{-2}	1.35 [1.17 – 1.57] × 10^{-2}	-	0.39 [0.34 – 0.44]

Data show values with 95% creditability intervals (CI) from the MCMCglmm models. If the 95% CIs of the random effect did not overlap with zero, this was interpreted as evidence for significant repeatability.

Table S4. Variance and repeatability in group (gr) and individual (id) behaviour across both trials of the cover context.

	Var group level	Var individual level	Var residual	R_C group	R_C individual
gr speed	0.79 [0.42 – 1.63]	0.00 [0.00 – 0.00]	0.97 [0.80 –1.18]	0.45 [0.34 – 0.58]	0.00 [0.00 - 0.00]
gr centre distance	2.75 [1.48 – 5.54]	0.00 [0.00 – 0.20]	3.16 [2.61 – 3.87]	0.47 [0.36 – 0.58]	0.00 [0.00 - 0.02]
gr polarisation	1.21 [0.35 – 3.65] × 10^{-3}	-	3.86 [2.38 – 6.47] × 10^{-3}	0.24 [0.13 – 0.36]	-
gr leadership structure	4.27 [1.91 – 9.78] × 10^{-4}	-	6.38 [3.96 – 10.89] × 10^{-4}	0.40 [0.32 – 0.48]	-
id leadership	-	1.02 [0.36 – 1.81] × 10^{-2}	0.24 [0.19 – 3.22] × 10^{-2}	-	0.30 [0.16 – 0.36]

Data show values with 95% creditability intervals (CI) from the MCMCglmm models. If the 95% CIs of the random effect did not overlap with zero, this was interpreted as evidence for significant repeatability.
